# Total myocardial revascularization for situs inversus totalis with dextrocardia: a case report

**DOI:** 10.1186/1752-1947-1-18

**Published:** 2007-05-05

**Authors:** Abbasali Karimi, Abbas SalehiOmran, Hossein Ahmadi, Parin Yazdanifard

**Affiliations:** 1 Department of Cardiothoracic Surgery, Tehran Heart Centre, Medical Sciences/University of Tehran, Tehran, Iran; 2 Department of Clinical Research, Tehran Heart Centre, Medical Sciences/University of Tehran, Tehran, Iran

## Abstract

We report our experience of two patients suffering from severe coronary artery disease and situs inversus totalis with dextrocardia. The surgeon, standing on the right side of the patients, performed coronary artery bypass grafting by harvesting the right internal mammary artery in lieu of the left one.

## Introduction

Dextrocardia, defined as the presence of a right-sided heart, can be mirror image or isolated. Situs inversus totalis with dextrocardia was first described by anatomist surgeon Marco Aurelio in 1643 [1]. The etiology of this anomaly, with an approximate prevalence of 1–2/10,000 normal population, has hitherto eluded the medical community, although it is thought to be autosomal recessive. The rate of atherosclerotic heart disease in people with this condition is similar to the general population [2]. We present our experience with 2 cases of situs inversus totalis with dextrocardia. The patients, who had coronary artery disease, underwent coronary artery bypass grafting (CABG) with the surgeon standing on the right side of the patients and the right internal mammary artery (RIMA) harvested for bypass grafting instead of the left internal mammary artery (LIMA).

### Case A

A 58-year-old woman, who had experienced myocardial infarction (MI) one year previously, presented with post MI angina. Already diagnosed with situs inversus totalis with dextrocardia, she had a history of diabetes, hypertension, hyperthyroiditis and hypercholesterolemia. Coronary angiography showed three-vessel disease with significant stenosis in the proximal and mid portions of the left anterior descending (LAD) artery, distal portion of the left circumflex (LCX) artery and proximal and mid portions of the right coronary artery (RCA). Left ventricular ejection fraction (LVEF) was about 60%. Elective CABG was considered for the patient. A median sternotomy revealed that the heart occupied exactly the mirror image of its normal position. The saphenous vein and RIMA were harvested by the surgeon, standing on the right side of the patient instead of the left side. Cannulation was performed, and a routine antegrade cardioplegia was administered.

**Figure 1:**
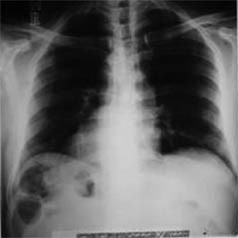
Chest X-Ray of patient No-1.

The RIMA and saphenous vein grafts were grafted on the LAD, obtuse marginatus (OM) and RCA, respectively. Cardiopulmonary bypass (CPB) time and aortic cross-clamp time were 51 and 80 minutes, respectively. The patient was weaned from CPB in normal sinus rhythm and without inotropic support. The postoperative course being smooth and uneventful, the patient was discharged on the 8^th ^postoperative day in good condition. Figure 1 shows the angiographic features of significant stenosis in the mid portion of the right coronary artery (RCA) of the patient.

### Case B

A 46-year-old man, already diagnosed with situs inversus totalis with dextrocardia and coronary artery disease, presented with signs and symptoms of chronic stable angina. He was a cigarette smoker, and had suffered myocardial infarction about two years before. After coronary angiography, showing three-vessel disease with significant stenosis in the proximal part of the LAD, circumflex and right coronary arteries and an approximate ejection fraction of 50–60%, the patient underwent elective CABG. A median sternotomy was performed, revealing the heart occupying exactly the mirror image of its normal position. Similar to the previous case, the surgeon stood on the right side of the patient to harvest the saphenous vein and RIMA. Cannulation was carried out, and an antegrade cardioplegia was administered routinely. The RIMA and three vein grafts were grafted on the LAD, OM, diagonal and distal portion of RCA, respectively. CPB and aorta cross-clamp times were 93 and 57 minutes, respectively. The patient was weaned from CPB without inotropic support in normal sinus rhythm. The patient had an uneventful postoperative course and was discharged 6 days after surgery.

## Discussion

Not only is dextrocardia with situs inversus totalis a rare finding, there are also only a few reports of this anomaly in tandem with coronary artery disease and CABG in the existing literature [2-4]. The rate of coronary artery disease in those with this anomaly is similar to that in the normal population [2].

There are two significant points in our method that merit due attention. First, in contrast to previous studies reporting the surgeon standing on the left side of the patient [3], our surgeon performed uneventful operations while standing on the right side of the patient. Second, the RIMA, rather than LIMA, was harvested for grafting on the left anterior descending artery.

In conclusion, mirror-image anatomy does not pose an unusual technical challenge in total myocardial revascularization and surgeons can perform surgery without difficulty while standing on the right side of the patient.

## Abbreviations

Coronary artery bypass grafting (CABG), Right internal mammary artery (RIMA), Left internal mammary artery (LIMA), Myocardial infarction (MI), Left anterior descending (LAD), Right coronary artery (RCA), Left ventricular ejection fraction (LVEF), Left circumflex(LCX), Obtuse marginatus (OM), Cardiopulmonary bypass (CPB).

## Competing interests

The author(s) declare that they have no competing interests.

## Authors' contributions

**AK **carried out the surgery and was directly involved in the conception, design and drafting of the manuscript. **AS **and **HA **participated in the surgery; they also gave critical comments on the results and participated in planning and coordinating the study. **PY **collaborated in the design of the study and was directly involved in drafting and revising the manuscript. All the authors read and approved the final manuscript.
